# Major Adverse Cardiovascular Event Outcomes in Patients With Obesity and Type 2 Diabetes Undergoing Medical Weight Management

**DOI:** 10.7759/cureus.94070

**Published:** 2025-10-07

**Authors:** Nirjhar Dutta, Katelyn M Tessier, Jenna Langer, Tasma Harindhanavudhi, Shalamar D Sibley, Charles Billington, David Vock, Eric S Wise, Eric M Bomberg, Carolyn Bramante

**Affiliations:** 1 Division of General Internal Medicine, Department of Medicine, University of Minnesota, Minneapolis, USA; 2 Masonic Cancer Center, Biostatistics Core, University of Minnesota, Minneapolis, USA; 3 Division of Endocrinology, Diabetes, and Metabolism, Department of Medicine, University of Minnesota, Minneapolis, USA; 4 Department of Endocrinology, Diabetes, and Metabolism, Minneapolis Veterans Affairs Health Care System, Minneapolis, USA; 5 Division of Biostatistics, University of Minnesota School of Public Health, Minneapolis, USA; 6 Department of Surgery, University of Minnesota School of Medicine, Minneapolis, USA; 7 Division of Pediatric Endocrinology, Department of Pediatrics, University of Minnesota Masonic Children's Hospital, Minneapolis, USA; 8 Division of Geriatrics, Palliative, and Primary Care, Department of Medicine, University of Minnesota, Minneapolis, USA

**Keywords:** bariatric & metabolic surgery, major adverse cardiovascular events (mace), multidisciplinary weight management, obesity and diabetes, weight management drugs

## Abstract

Objective: We assessed the impact of medical weight management (MWM; lifestyle modification ± obesity medications) on major adverse cardiovascular events (MACE) compared to metabolic and bariatric surgery (MBS) and usual care (UC).

Methods: We retrospectively analyzed electronic health records of adults with body mass index (BMI) ≥35 kg/m² and type 2 diabetes mellitus (T2D) at an academic health center from 2010 to 2021. The MWM group was propensity score matched on common confounders 1:1 (versus MBS) and 1:5 (versus UC). The primary outcome was a six-component MACE (all-cause mortality, coronary artery events, cerebrovascular events, heart failure, atrial fibrillation, and nephropathy).

Results: Among 2,100 patients (300 MWM, 300 MBS, and 1,500 UC), baseline characteristics were similar among groups. During a median 3.2-year follow-up (range 0-11), the adjusted hazard ratio (aHR) for MACE for MWM versus MBS was 1.61 (0.98-2.65, p=0.06); for MBS versus UC, aHR 0.66 (0.43-1.02, p=0.06); and there was no difference in MWM versus UC, aHR 1.07 (0.77-1.49, p=0.68).

Conclusions: No statistically significant differences in MACE risk were found between those receiving MWM versus UC; there was a trend towards fewer MACE events in those receiving MBS. These findings must be validated in future studies, given that more effective weight loss medications (e.g., semaglutide, tirzepatide) were not available.

## Introduction

Obesity is a chronic disease affecting over 42% of adults in the United States [1]. The pathophysiology of obesity is complex and multifactorial. Excess visceral adiposity in obesity is thought to lead to a chronic mild inflammatory state, mediated by free fatty acid-induced reactive oxygen species, upregulation of pro-inflammatory adipokines, and downregulation of anti-inflammatory adipokines, leading to insulin resistance, atherosclerosis, impaired angiogenesis, fibrosis, and appetite dysregulation. This, in turn, leads to the development of several diseases, including type 2 diabetes mellitus (T2D), coronary artery disease, and non-alcoholic fatty liver disease. 

The first-line treatment of obesity is health behavior and lifestyle treatment (HBLT), consisting of dietary modifications, physical activity, and behavioral counseling. Such programs have been shown to lead to 7% to 10% total body weight (TBW) loss in the first year and over 5% sustained weight loss at 10 years [2]. Obesity medications (OMs) can be used as an adjunct to HBLT given their roles in modifying the underlying biological drivers of obesity. Currently, six OMs are FDA-approved for long-term use for obesity management in adults: orlistat, phentermine/topiramate, naltrexone/bupropion, liraglutide, semaglutide, and tirzepatide. Several others are used off-label, including phentermine (≥3 months), topiramate, naltrexone, bupropion, and exenatide [3,4]. While older OMs can lead to 5% to 10% TBW loss, newer agents like semaglutide, which is a long-acting glucagon-like peptide 1 (GLP1RA) receptor agonist, and tirzepatide, which is a long-acting GLP-1 and gastric inhibitory polypeptide (GIP/GLP1RA) co-agonist, can achieve about 15% to 20% of TBW loss over placebo at more than one year follow-up [5-7]. 

Patients with class 2 or 3 obesity (defined as body mass index (BMI) ≥ 35 kg/m² (≥ 27.5 for Asians)), or with class 1 obesity (defined as BMI ≥ 30 kg/m² (≥ 25 for Asians)) and obesity-related complications and co-morbidities, may be candidates for metabolic/bariatric surgery (MBS), of which the most commonly performed procedures are Roux-en-Y gastric bypass (RYGB) and sleeve gastrectomy (SG) [8]. MBS has been shown to lead to the greatest TBW loss on average, with SG typically leading to ~15% and RYGB leading to ~25% TBW loss at the five-year mark, with both leading to remission of T2D in over 50% of patients [9]. Further, MBS appears to reduce the risk of mortality and major adverse cardiovascular events (MACE), including heart attack and stroke, in patients with T2D and obesity [10-12]. 

No HBLT interventions and, until the arrival of newer GLP1-RAs (e.g., semaglutide), no OM has shown meaningful reductions in MACE risk in patients with obesity and without T2D; although meta-analyses of randomized controlled trials (RCTs) evaluating GLP1RA use in patients with T2D have shown statistically significant MACE reductions [2, 13-15]. The impact of medical weight management (MWM), which includes HBLT with or without OMs, on MACE is largely unknown, especially in a “real-world” clinical setting where patient populations and outcomes may differ compared to those seen in randomized trials [16].

Using electronic health record (EHR) data, we sought to compare the impact of MWM, MBS, or neither (usual care (UC); no history of MBS, OM use, or MWM encounters) on MACE risk. We hypothesized that MACE events would be reduced between those receiving MWM compared to UC, reduced between those who underwent MBS compared to UC, and similar between those receiving MWM and MBS.

## Materials and methods

Study design and participants

We performed a retrospective cohort study using EHR data of adults seen in a U.S. Midwestern academic health center-based healthcare setting from 2010 to 2021. This study was approved by the Institutional Review Board (IRB) of the University of Minnesota, Minneapolis, MN (approval number: STUDY00006410). Inclusion criteria included adults (aged ≥ 18) with class 2 or 3 obesity (defined as BMI ≥ 35 kg/m²) and T2D (defined as hemoglobin A1c > 6.5 or International Classification of Diseases (ICD) code for T2D and exclusion of T1D [17,18]) who did not opt out of having their EHR reviewed for the purposes of research via the Consent for Services form that all patients complete (at our institution, the opt-out rate is typically 1-2%). A BMI cut-off of ≥35 kg/m² was chosen to match the typical criteria for qualifying to undergo MBS [8]. We excluded patients with active cancer and heart failure with reduced ejection fraction, and solid organ transplant patients were excluded (liver, heart, lung), consistent with published literature [10]. Patients were categorized into three groups, which were as follows: (1) MWM: The MWM group included those seen ≥2 times in our MWM clinics and without a history of MBS. All patients seen in our MWM clinics receive HBLT with or without OMs. OMs prescribed included liraglutide, exenatide, bupropion/naltrexone (prescribed either as a combination or as separate components), orlistat, phentermine/topiramate (prescribed either as a combination or as separate components), lorcaserin, and sibutramine for any amount of time. (2) MBS: The MBS group included those with a history of MBS procedures (RYGB, SG, duodenal switch, or adjustable gastric banding) regardless of establishment with our MWM clinics or having OMs prescribed. (3) UC: The UC group included patients who were neither seen in our MWM clinics nor prescribed OMs, nor had any history of MBS; however, they had been seen in our healthcare system for any outpatient encounter.

Variables

All variables were derived from a review of EHR data. ICD and Current Procedural Terminology (CPT) codes [19] were used, and the variables were mapped to the Unified Medical Language System identifier, consistent with prior literature (see Appendix A for diagnosis and procedure codes) [10]. Our primary outcome was a six-component MACE (composite of all-cause mortality, coronary artery events, cerebrovascular events, heart failure, atrial fibrillation, and nephropathy) incidence from the index date [13]. Data quality checks were done by at least one investigator in about 10% of the sample size by manual chart review, and values far outside the normal range were omitted or corrected.

Statistical analyses

The MWM group was propensity score (PS) matched to the MBS and UC groups in 1:1 and 1:5 ratios, respectively, using the nearest-neighbor method from a logistic regression model with a logit link function. Covariates in the PS model were determined a priori and included index date, age at index date, sex, median household income, BMI (class 2 or 3 obesity), use of insulin, and presence of diabetes-related end-organ complications (coronary artery disease/myocardial infarction, ischemic stroke, peripheral arterial disease, diabetic neuropathy, and nephropathy), all variables thought to be confounders [10,20].

Demographics and outcomes are summarized using descriptive statistics for all PS-matched patients and by treatment group and compared using Student’s t-tests, Wilcoxon rank-sum, Chi-square, or Fisher’s exact tests (as appropriate). Outcomes were defined from an index date (MBS: procedure date; MWM: first MWM visit; UC: random visit in the healthcare system) and were censored at the date of last follow-up in the healthcare system through the end of the defined study period if the outcome did not occur. Individual MACE components (e.g., all-cause mortality, coronary artery events) were defined using competing risks; therefore, if a patient died prior to having the individual MACE component, they were censored at the death date. This strategy is in line with previously published literature [10].

Absolute standardized mean differences are summarized to show the benefit of PS matching. Cumulative incidence over eight years for our 6-component MACE was performed using Kaplan-Meier (KM) curves. Cox proportional hazard regression models were used, with and without adjustment of PS-matched covariates, and hazard ratios (HR) and 95% confidence intervals (CI) were obtained. Models were adjusted for index date, sex, age at index date, continuous BMI, insulin use, presence of diabetes-related end-organ complications, and median household income. Cumulative incidence over eight years for individual MACE components was performed using the cumulative incidence function (CIF) curves. For individual components of the six-component MACE (aside from all-cause mortality), Fine and Gray regression models for competing risks were used, with and without adjustment of PS-matched covariates, and sub-distribution hazard ratios and 95% CIs were obtained. Cumulative incidence estimates and 95% CIs were obtained for each outcome and treatment group at years one, four, and eight.

To determine if a reduction in MACE was related to the amount of weight loss, we performed a subgroup analysis of patients achieving ≥15% TBW loss, as previous studies have shown that to be the average amount of TBW achieved with MBS [10]. To account for possible discrepancies in MACE reporting in the EHR, a subgroup analysis was done on patients with established primary care in our healthcare system, with the presumption that those with primary care based in our system should have more complete reporting of MACEs in the EHR. We defined having established primary care in the health system as the presence of ≥2 visits to a primary care clinic.

To separate out the possible TBW effect of OMs on patients with a history of MBS, a subgroup analysis was done on those with a history of MBS who had never received OMs. Obesity is a chronic disease, and weight loss maintenance requires long-term treatment with frequent clinic visits. There is a study that showed that patients with < 10 clinic visits lost less weight than those with > 10 clinic visits; however, only 2% of the population had achieved > 10 visits [21]. To evaluate the effects of long-term MWM clinic follow-up on MACE, we performed a subgroup analysis among patients with >5 MWM clinic visits, as that is likely to show adherence with the MWM clinic for a reasonably long duration while still having a meaningful sample size. 

For the overall cohort, outcomes were defined from the index date (MBS group: date of MBS; MWM group: date of first MWM visit; UC: date of random visit in the healthcare system) and were censored at the date of last follow-up in the healthcare system through the end of the defined study period if the outcome did not occur. Individual components of MACE were defined using competing risks; therefore, if a patient died prior to having the individual component of MACE, they were censored at the death date. 

Demographics and outcomes were summarized using descriptive statistics for all PS-matched patients and by treatment group and compared using Student’s t-tests, Wilcoxon rank-sum, chi-square, or Fisher’s exact tests, when appropriate. All reported p-values are two-sided, and a significance level of 0.05 was used. Statistical analyses were performed using R version 4.1.2 (The R Core Team, R Foundation for Statistical Computing, Vienna, Austria) [22]. 

## Results

Among 2,100 patients included in our analyses (300 MWM, 300 MBS, and 1,500 UC), baseline characteristics were similar among the three propensity-matched cohorts (1,374 (66%) female; median age 50 years (range 18-78 years); 1,468 (69.9%) BMI ≥40 kg/m²) (Table 1). Median BMI was statistically significantly higher for the MWM group compared to the UC or MBS group. All groups achieved TBW reduction during a median 3.2-year follow-up (range 0-11 years), ranging from a median (range) of 5.1% for UC to 7.5% for MWM and 23.8% for MBS. Mean and standard deviation (SD) for TBW loss were 6.9% (SD 8.4) in the UC group, 24% (SD 11) in the MBS group, and 9.2% (SD 8.1) in the UC group. Among the group, 60% (180) of patients in the MWM group, 96% (288) in the MBS group, and 77% (1155) in the UC group had primary care established in our healthcare system, and 86% (258) of those in the MWM group and 58% (174) in the MBS group were on OM(s) (Table 1). MBS procedures consisted of 50% (150) SG, 45% (135) RYGB, 4% (12) duodenal switch, and 1% (3) adjustable gastric banding. In the MWM group, the median number of MWM clinic visits was four (35% (105) had two visits, 39% (117) had three to five visits, and 27% (81) had more than five visits). 

**Table 1:** Baseline characteristics ^1^P-value is for Student’s t-tests for continuous variables and chi-square tests for categorical variables; ^3^Not mutually exclusive; ^4^TS: chi-squared test statistics; DF: chi-squared test degrees of freedom. MWM: medical weight management group; UC: usual care group; MBS: metabolic/bariatric surgery group

Variable	UC (N=1,500)	MBS (N=300)	MWM (N=300)	P-value^1^	Chi-square^4^
Female sex, n (%)	969 (64.6%)	212 (70.7%)	194 (64.7%)	0.12	TS 4.17, DF 2
Age (years), Median (range)	50.2 (18.0, 77.6)	48.2 (17.3, 72.7)	50.0 (18.8, 72.1)	0.37	
BMI (kg/m^2^), Median (range)	42.1 (35.0, 77.8)	42.8 (35.0, 74.6)	43.5 (35.0, 87.7)	<0.001	
BMI (kg/m^2^), Mean (standard deviation)	43.0 (5.7)	44.6 (7.3)	46.1 (10.1)	<0.001	
BMI (kg/m^2^), Categorical, n (%)				0.88	TS 0.26, DF 2
35-39.9	449 (29.9%)	89 (29.7%)	94 (31.3%)		
≥ 40	1051 (70.1%)	211 (70.3%)	206 (68.7%)		
Insulin use, n (%)	405 (27.0%)	90 (30.0%)	80 (26.7%)	0.54	TS 1.22, DF 2
Diabetes-related end-organ complications, n (%)	133 (8.9%)	32 (10.7%)	29 (9.7%)	0.59	TS 1.04, DF 2
Obesity medications^3^, n (%)	N/A	168 (56.0%)	258 (86.0%)	-	
Liraglutide	N/A	45 (15.0%)	119 (39.7%)	-	
Exenatide		36 (12.0%)	52 (17.3%)	-	
Bupropion		58 (19.3%)	68 (22.7%)	-	
Naltrexone		19 (6.3%)	48 (16.0%)	-	
Bupropion/Naltrexone		6 (2.0%)	11 (3.7%)	-	
Phentermine		43 (14.3%)	71 (23.7%)	-	
Topiramate		70 (23.3%)	191 (63.7%)	-	
Phentermine/Topiramate		5 (1.7%)	15 (5.0%)	-	
Other		10 (3.3%)	18 (6.0%)	-	
Metabolic/Bariatric surgery, n (%)	N/A		N/A	-	
Roux-en-Y gastric bypass	N/A	125 (45.1%)	N/A	-	
Sleeve gastrectomy		138 (49.8%)			
Other		14 (5.1%)			
Median household income, USD (range)	61758 (12292, 185481)	61197 (16461, 145995)	60322 (13950, 147039)	0.59	

**Figure 1:**
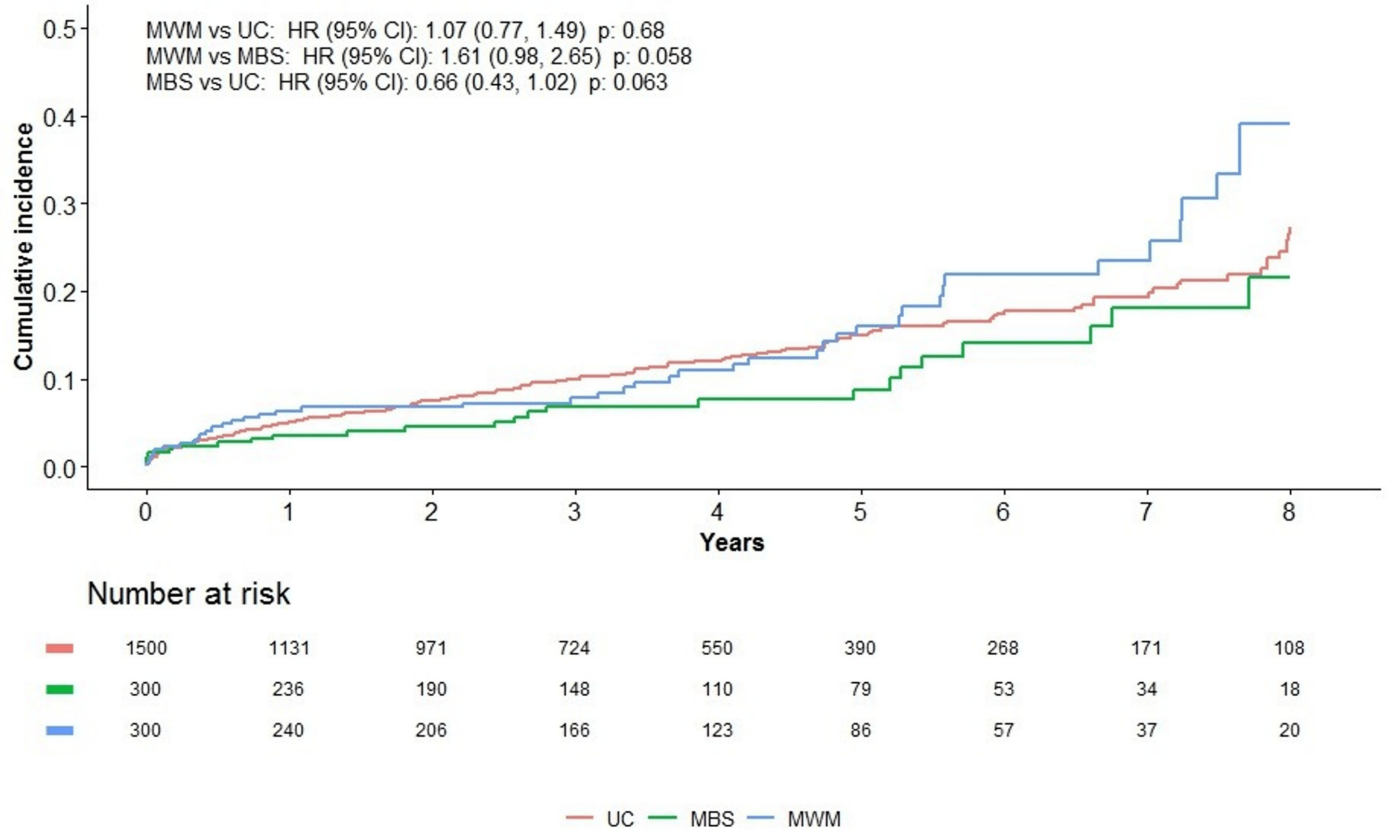
Eight-year cumulative incidence estimates for six-component major adverse cardiovascular event (MACE) outcomes in medical weight management (MWM) vs. metabolic/bariatric surgery (MBS) vs. usual care (UC). Eight-year cumulative incidence estimates were derived using the Kaplan-Meier method. Hazard ratio (HR) and 95% confidence intervals (CI) were derived using Cox proportional hazard regression adjusted for index date, sex, age at index date, continuous BMI, insulin use, presence of diabetes-related end-organ complications, and median household income.

For the composite six-component MACE and individual components of the MACE, outcomes are shown in Table 2. Fifteen percent (45) of patients in the MWM group, 8% (24) in the MBS group, and 12% (180) in the UC group experienced a six-component MACE during the study period. Cumulative incidence of the six-component MACE at the eight-year follow-up was lowest in the MBS group (0.22) compared to the UC (0.27) and MWM (0.39) groups. Yearly cumulative incidence estimates for each group are shown in Figure 1. Adjusted hazard ratio (aHR) for MACE events for MWM versus MBS was 1.61 (0.98-2.65, p=0.058), favoring MBS; for MBS versus UC, aHR was 0.66 (0.43-1.02, p=0.063), favoring MBS; and for MWM versus UC, aHR was not significantly different (p=0.68).

**Table 2:** Adjusted regression estimates for major adverse cardiovascular event (MACE) outcomes Cumulative incidence estimates for six-component MACE and all-cause mortality are derived using the Kaplan-Meier method. The cumulative incidence function (CIF) was used for the remaining outcomes due to competing risks. Hazard ratio (HR) estimates and 95% confidence intervals (CI) for six-component MACE and all-cause mortality are derived using Cox proportional hazard regression. Fine and Gray regression models were used for the remaining outcomes due to competing risks. Models were adjusted for index date, sex, age at index date, continuous BMI, insulin use, presence of diabetes-related end-organ complications, and median household income. MWM: medical weight management group; UC: usual care group; MBS: metabolic/bariatric surgery group

Outcome	MWM vs. UC		MWM vs. MBS		MBS vs. UC		Cumulative incidence estimate at the eight-year follow-up		
	HR (95% CI)	P-value	HR (95% CI)	P-value	HR (95% CI)	P-value	UC	MBS	MWM
6-component MACE	1.07 (0.77, 1.49)	0.68	1.61 (0.98, 2.65)	0.06	0.66 (0.43, 1.02)	0.06	0.27 (0.22, 0.32)	0.22 (0.11, 0.31)	0.39 (0.25, 0.51)
Individual MACE Components									
All-cause mortality	2.14 (0.37, 12.4)	0.40	1.74 (0.15, 19.7)	0.66	1.23 (0.13, 11.5)	0.86	0.01 (0.00, 0.03)	0.01 (0.00, 0.03)	0.01 (0, 0.02)
Coronary artery event	1.20 (0.69, 2.09)	0.52	2.08 (0.87, 5.00)	0.10	0.57 (0.26, 1.25)	0.16	0.07 (0.05, 0.10)	0.04 (0.02, 0.09)	0.15 (0.07, 0.26)
Cerebrovascular event	0.82 (0.34, 2.02)	0.67	1.25 (0.38, 4.15)	0.72	0.66 (0.24, 1.82)	0.42	0.04 (0.03, 0.06)	0.05 (0.02, 0.11)	0.08 (0.02, 0.19)
Heart failure	1.65 (0.36, 7.48)	0.52	-	-	-	-	0.01 (0.00, 0.02)	0	0.01 (0.00, 0.03)
Atrial fibrillation	0.89 (0.44, 1.77)	0.73	0.82 (0.33, 2.00)	0.66	1.08 (0.54, 2.18)	0.82	0.06 (0.04, 0.09)	0.10 (0.04, 0.19)	0.06 (0.03, 0.10)
Nephropathy	0.93 (0.5, 1.75)	0.83	1.22 (0.48, 3.12)	0.67	0.76 (0.36, 1.61)	0.48	0.139 (0.10, 0.19)	0.09 (0.03, 0.17)	0.14 (0.06, 0.25)

MACE outcomes in the subgroup analyses are shown in Table 3. It was noted that ≥15% TBW loss was achieved by 75% (225) of the MBS group, compared to only 18% (54) of the MWM and 12% (180) of the UC groups. Median TBW loss in this subgroup of patients that lost >15% TBW was as follows: 20.9% in MWM, 26.7% in MBS, and 20.6% in UC. Mean (SD) TBW loss in this subgroup that lost >15% TBW was as follows: 22.5% (5.7) in MWM, 27.7% (8.9) in MBS, and 22.4% (6.8) in the UC groups. Among this cohort, there were statistically significant differences in MACE risk between MWM and MBS (aHR 3.15, 95% CI 1.44-6.89, p=0.004) and between MBS and UC (aHR 0.3, 0.15-0.59, p<0.001), both favoring MBS. There was no statistically significant difference in MACE between those receiving MWM and UC (aHR 0.95, 0.45-2.02, p=0.893).

**Table 3:** Adjusted regression estimates for major adverse cardiovascular event (MACE) outcomes in subgroup analyses Models were adjusted for index date, sex, age at index date, continuous BMI, insulin use, presence of diabetes-related end-organ complications, and median household income. MWM: medical weight management group; UC: usual care group; MBS: metabolic/bariatric surgery group

	Sample size	MWM vs. UC		MWM vs. MBS		MBS vs. UC	
		HR (95% CI)	P-value	HR (95% CI)	P-value	HR (95% CI)	P-value
≥15% weight loss cohort	N = 54 for MWM N = 225 for MBS N = 173 for UC	0.95 (0.45-2.02)	0.89	3.15 (1.44-6.89)	0.004	0.3 (0.15-0.59)	<0.001
Primary care in the healthcare system	N = 180 for MWM N = 288 for MBS N = 1150 for UC	1.11 (0.74-1.65)	0.61	1.51 (0.88-2.59)	0.13	0.73 (0.47-1.14	0.17
MBS without obesity medications	N= 300 for MWM N = 132 for MBS N = 1500 for UC	N/A	N/A	1.91 (0.86-4.26)	0.11	0.56 (0.26-1.21	0.14
>5 MWM clinic visit	N = 80 for MWM N = 300 for MBS N = 1500 for UC	0.83 (0.48-1.43)	0.50	1.26 (0.66-2.41)	0.48	N/A	N/A
Individual Obesity Medications (only drugs with N > 50 in the MWM group shown)							
Liraglutide	N = 119 for MWM N = 45 for MBS N = 1500 for UC	0.99 (0.61, 1.59)	0.96	0.79 (0.34, 1.83)	0.59	1.25 (0.6, 2.6)	0.56
Exenatide	N = 52 for MWM N = 36 for MBS N = 1500 for UC	0.81 (0.42, 1.55)	0.53	1.42 (0.51, 3.95)	0.50	0.57 (0.25, 1.33)	0.19
Topiramate	N = 129 for MWM N = 49 for MBS N = 1500 for UC	1.06 (0.67, 1.68)	0.80	1.79 (0.61, 5.28)	0.29	0.59 (0.21, 1.64)	0.31
Phentermine-topiramate	N = 68 for MWM N = 22 for MBS N = 1500 for UC	0.76 (0.28, 2.09)	0.60	1.05 (0.12, 9.44)	0.96	0.72 (0.10, 5.26)	0.75

In terms of having primary care established within our healthcare system, there was no difference in MACE outcomes between those who did and did not have primary care established. 

Patients with a history of MBS and not prescribed OMs had similar outcomes compared to those who underwent MBS and were prescribed OMs, without statistically significant differences in MACE outcomes. Median TBW loss in patients with a history of MBS and not prescribed OMs was 24.7%, which is very similar to the overall MBS cohort’s median TBW loss of 23.8%. 

When evaluating specific OMs, there were no statistically significant differences in MACE outcomes among groups (Table 3, only drugs with N > 50 in the MWM group are shown).

Among patients seen in the MWM clinic more than five times, median TBW loss was 10.5%, compared to 6.1% among those with only two visits at the MWM clinic. Patients seen in the MWM clinic more than five times showed similar MACE risk compared to UC and to MBS, all without statistically significant differences.

## Discussion

Among 2,100 patients with class 2 or 3 obesity and T2D seen from 2010 to 2021 in a U.S.-based academic healthcare system, we found no statistically significant differences in MACE risk between those seen in MWM clinics and those receiving UC over a median 3.2-year follow-up. We did find a trend showing that those who underwent MBS had a lower risk of MACE events compared to those receiving MWM or UC (both p=0.06), the former of which is a novel finding and the latter aligned with previous literature [10-12]. 

There may be multiple reasons behind our findings of a trend towards reduced MACE in those with a history of MBS compared to those in the MWM or UC groups. In our study, the MBS group experienced greater TBW compared to the MWM and UC groups, and it may be that MACE risk reduction is a function of weight loss. In a large cohort study of patients with obesity and T2D, it was previously shown that MACE outcomes improve after about 10% TBW loss among adults who underwent MBS [23]. That said, in our subgroup analyses of patients who experienced ≥15% TBW loss, we again found MACE risk reduction in the MBS compared to the MWM and UC groups. Therefore, it may also be possible that MACE reduction following MBS may not be entirely weight loss dependent but also due to metabolic changes that occur following MBS. For example, a large cohort study of patients with obesity and T2D, 1,223 with MBS and 5,978 non-surgical controls, over almost five years of follow-up, showed that while patients with a history of MBS required at least 10% TBW loss to see improvement in MACE risk, those without MBS needed >20% TBW loss, suggesting that metabolic and endocrine factors independent of weight loss may play a role in reducing MACE outcomes [23].

In our study, when we evaluated OMs separately, exenatide and phentermine/topiramate showed a trend towards reduction in MACE compared to UC; however, this was not statistically significant, and the sample size was small (Table 3). In randomized trials, liraglutide but not exenatide has been shown to reduce MACE in patients with T2D [24, 25]. It is possible that the weight loss or endocrine changes required to reduce MACEs are not readily achievable with less potent OMs such as phentermine monotherapy, topiramate monotherapy, exenatide, and liraglutide, the primary OMs evaluated in this study.

Not surprisingly, those in the MBS group achieved greater TBW loss (23.8%) compared to those in the MWM (7.5%) or UC (5.1%) groups. We consider the finding that those in the UC care group experienced TBW loss rather than weight maintenance or increase as a surprise that may warrant further investigation. One limitation of our study is that records from outside healthcare settings were not reliably available in the EHR, and it could have been that some were receiving HBLT, or even OMs or other medications associated with weight loss (prescribed outside our system and not manually entered into the patient’s medication list), from outside providers not captured in the EHR. It may also have been that due to our population consisting of adults with T2D and with class 2 and 3 obesity, as research has previously shown that TBW may be greater in this group compared to those with T2D and lower BMI. For example, a previously published retrospective cohort study of patients with class 2 or 3 obesity showed ~ 25% lost ≥5% TBW during a five-year follow-up; however, the median weight change for the population was a net gain of 2.5% TBW [26]. The amount of weight loss achieved in the UC compared with the MWM group may explain why we found no statistically significant difference in MACE outcome between the MWM vs. UC groups.

To determine if the degree to which follow-up in the MWM is associated with MACE risk, we performed a subgroup analysis only within the MWM group who were seen in clinic more than five times (Table 3). While those with more than five visits to the MWM did not see a reduction in MACE events compared to UC, they did experience a greater TBW loss compared to those with fewer MWM visits. This is an interesting finding that requires a longer follow-up in a larger sample because, if true, this would suggest that patients who are compliant with the MWM clinic over a long period achieve greater TBW loss. Further, in our subgroup analysis among patients with a history of MBS, comparing those prescribed OMs to those not, results were similar, suggesting that the MACE risk reduction from MBS may not be dependent on OMs. 

We additionally note that the MWM group had a somewhat higher baseline median BMI (43.5 kg/m²) compared to the MBS (42.8 kg/m²) and UC (41.1 kg/m²) groups. This finding is not entirely surprising, as it may be that some patients seen in MWM have a higher comorbidity burden and therefore may be less suitable as surgical candidates for MBS. If such were the case, it is possible that the difference in risk between MBS and MWM was not related to the surgery but rather due to those in the MWM group having more obesity-related comorbidities. 

Our results must be interpreted within the context of limitations. For one, as this was an observational study, only associations can be determined, rather than causation. This study also relied on EHR data, which has inherent limitations as it relies on clinical providers to enter data accurately and completely [27]. We did perform data quality checks on 10% of the sample size with manual chart review, and values far outside the normal range were omitted or corrected; this happened in about 1% to 2% of cases. There may be missing data due to the fact that patients may also be followed for care outside of our healthcare system, and it is possible that all data from outside healthcare systems is not linked to our dataset. However, this may be the case for all groups similarly [28]. Given that our results come from a single urban center located in the Midwestern portion of the U.S., it is possible that the results may not be generalizable to other populations, including those in rural settings, consisting of other ancestries, or where access, use, and availability of OMs and/or MBS may differ [29]. Moreover, this study only had a median of 3.2 years of follow-up, which may not have been long enough for MACE development, and longer-term studies are needed. Additionally, our analyses of the effects of particular OMs on MACE risk are more limited due to small sample sizes.

Importantly, we note that in our study cohort, there were no patients on newer GLP1RAs (e.g., semaglutide) or GIP/GLP1RAs (e.g., tirzepatide) given the study period (2010-2021). Semaglutide first received FDA approval for the treatment of T2D in adults in 2017 and obesity in 2021, while tirzepatide was first FDA approved for T2D in 2022 and obesity in 2023. In the SELECT trial of 17,603 adults with overweight and obesity and without T2D but with pre-existing cardiovascular disease, the HR for cardiovascular events was 0.80 (0.72-0.90) in the semaglutide group compared to placebo over an approximately three-year follow-up period [15]. Similarly, tirzepatide has been shown to decrease atherosclerotic cardiovascular disease (ACVD) risk scores in a randomized trial, and meta-analyses of retrospective studies have shown it to reduce all-cause mortality and adverse cardiovascular and renal outcomes in patients with T2D [30, 31]. Therefore, it may be that future observational cohort studies involving newer GLP1-RA/GIP/GLP1-RA-containing medications may show greater effects on MACE risk. However, it may also be that MACE risk reduction with these newer medications may still be less than with MBS.

Multicenter retrospective and prospective studies with longer durations and larger sample sizes are needed, particularly with the FDA approval of more efficacious OMs, which were not available during this study period. Further evaluation of MACE outcomes following other procedures is needed, including intragastric balloon therapy, aspiration therapy, and endoscopic SG (ESG). For example, ESG has been shown to reduce TBW by ~ 15%, sustained at five years, and with improvements in HbA1c and fatty liver disease; however, MACE outcomes following this procedure are unknown [32-34]. 

## Conclusions

Among those with class 2 or 3 obesity and T2D, we found no statistically significant differences in MACE risk between those receiving MWM versus UC or MWM versus MBS; however, there was a trend towards those in the MBS group having fewer MACE events compared to those receiving MWM or UC. Longer-duration, multi-center studies are needed to validate these findings, especially considering more effective weight loss medications, including those with cardioprotective effects (e.g., semaglutide, tirzepatide), were not available during the study period.

## Appendices

Appendix A 

**Table 4:** Diagnosis and procedure codes Sources: [10, 17-19]

Diagnosis	ICD / CPT Code
Atrial fibrillation	ICD9: 427.31 ICD10: i48.0, i48.1, i48.2, i48.91 CPT: 93650, 93653, 93656, 93657
Cancer	ICD9: 140-209 ICD10: C00-C96, D03, D3A, D45
Cerebrovascular event	ICD9 (diagnoses): 433.X1, 434.X1, 436.0, 430.X, 431.X ICD9 (procedure): 38.12, 0.61, 0.63 CPT-4: 37215, 37216, 0075T, 0076T, 35301, 37205, 37206
Coronary artery disease	ICD9 (diagnoses): 410.X, 411.X, 414.X ICD9 (procedure): 36.01, 36.02, 36.03, 36.05, 36.06, 36.07, 36.10, 36.11, 36.12, 36.13, 36.14, 36.15, 36.16, 36.17, 36.19, 36.31, 36.32, 36.33, 36.64 CPT-4: 92982, 92984, 92995, 92996, 92980, 92981, 33510, 33511, 33512, 33513, 33514, 33516, 33517, 33518, 33519, 33521, 33522, 33523, 33530, 33533, 33534, 33535, 33536, 93539, 93540
Type 1 Diabetes Mellitus	ICD9: 250.01, 250.03, 250.11, 250.13, 250.21, 250.23, 250.31, 250.33, 250.41, 250.43, 250.51, 250.53, 250.61, 250.63, 250.7, 250.73, 250.81, 280.83, 250.91,250.93 ICD10: E10 A1c > 6.5: test code 4548-4 and result >6.5
Type 2 Diabetes Mellitus	ICD9: 250.00, 250.02, 250.10, 250.12, 250.20, 250.22, 250.30, 250.32, 250.40, 250.42, 250.50, 250.52, 250.60, 250.62, 250.70, 250.72, 250.80, 280.82, 250.90, 250.92 ICD10: E11 A1c > 6.5: test code 4548-4 and result >6.5
Diabetic neuropathy	ICD9: 250.6, 357.2
Heart failure	ICD9: 428.0, 428.1, 428.20, 428.21, 428.22, 428.23, 428.30, 428.31, 428.32, 428.33, 428.40, 428.41, 428.42, 428.43, 428.9 ICD10: I50
Ischemic stroke	ICD-9: 433.X1, 434.X1 ICD-10: i63.X and i63.XX and i63.XXX
Liver, lung, heart transplant	ICD9: V42.1, V42.6, V42.7, V42.83, V42.84 ICD10: Z94.1-4
Myocardial infarction	ICD-9: 410.X, 410.XX ICD-10: i21.X and i21.XX, i22.X and i22.XX, i23.X and i23.XX
Nephropathy	>= two (2) measures of eGFR less than 60 mL/min separated by at least 90 days without any intervening values >= 60 mL/min. The eGFR is approximated using MDRD equation.
Peripheral vascular disease	ICD9: 440.20, 440.21, 440.22, 440.23, 440.24, 440.29, 440.31, 440.32, 443.81, 443.89, 443.9, 447.1, 249.7, 250.7
